# Unsafe storage of household medicines: results from a cross-sectional study of four-year-olds from the 2004 Pelotas birth cohort (Brazil)

**DOI:** 10.1186/s12887-019-1597-1

**Published:** 2019-07-12

**Authors:** Delba Fonseca Santos, Marysabel Pinto Telis Silveira, Aline Lins Camargo, Alicia Matijasevich, Iná Silva Santos, Aluísio J. D. Barros, Andréa Dâmaso Bertoldi

**Affiliations:** 1 Universidade Federal dos Vales do Jequitinhonha e Mucuri (UFVJM), Diamantina, MG Brazil; 2 Programa de Pós-Graduação em Epidemiologia e Instituto de Biologia, Departamento de Fisiologia e Farmacologia, Universidade Federal de Pelotas (UFPel), Rua Marechal Deodoro, 1160, Centro CEP 96020-220, Pelotas, RS Brazil; 3 Universidade Federal de Ciências da Saúde de Porto Alegre (UFCSPA), Porto Alegre, RS Brazil; 4 Departamento de Medicina Preventiva, Faculdade de Medicina FMUSP, Universidade de São Paulo, São Paulo, SP Brazil; 5 Programa de Pós-Graduação em Epidemiologia, Universidade Federal de Pelotas (UFPel), Pelotas, RS Brazil

**Keywords:** Medication storage, Pharmaceutical preparations, Accident prevention, Child health, Cohort studies

## Abstract

**Background:**

Unintentional child poisoning represents a significant public health problem across the globe, placing a substantial burden on health services emergency departments. Around the world, every year, thousands of children die as a result of physical injuries, most of which involve children under 5 years old. Medicines are the main products involved in poisoning, and children under 5 years old are the most vulnerable age group. The objective of this study was to measure the prevalence of unsafe storage of medicines in households with a 4-year-old child.

****Methods**:**

We used data from the follow-up of 4-year-old in the 2004 Pelotas Birth Cohort Study in Brazil (*N* = 3799). “Unsafe storage” was considered present when medicines were stored unlocked and within reach of children (at a height below the eye level of the average adult). Independent variables included maternal and family socioeconomic and demographic characteristics and the child’s health care. All information was collected during household interviews with the mothers using a standardized questionnaire. The overall prevalence rate with a 95% confidence interval (95% CI) and the prevalence associated with various independent variables were determined.

****Results**:**

The storage of medicines in unlocked areas was reported by 80.9% of the mothers, and, within reach of children for 26.5%. The overall prevalence rate of unsafe storage of medicines was 21.4% (20.1–22.7%). The main storage locations used were the kitchen (57.0%) and bedroom (53.3%).

**Conclusions:**

The results indicate that medicines were unsafely stored in a 21.4% number of homes, which can contribute to the vulnerability of children to poisoning from medicines. To minimize this risk, education about the safe storage of medicines should be reinforced by health professionals.

## Background

Unintentional child poisoning represents a significant public health problem globally, placing a substantial burden on health services emergency departments [1]. Every year, thousands of children around the world die as a consequence of physical injuries, most of them involving children under 5 years old [2]. Childhood injury prevention is considered a public health priority [3, 4]. Most domestic accidents leading to injuries in children are caused by falls, cuts, burns [5], and poisoning [6].

Medicines are the main type of products involved in accidental poisoning, and children under 5 years are the most vulnerable age group [7]. Today, there are more medicines at home than ever before, increasing the risk for poisoning. However, the vast majority of childhood poisoning incidents may be prevented [8]. Strategies to prevent this type of injury include changes in the packaging, labels and instruction materials of medicines, as well as the dissemination of knowledge about the type of medicines most frequently involved in poisoning, the circumstances in which poisonings occur and estimated underreporting rates.

Previous studies have shown that factors associated with childhood poisoning include the low educational level of the parents, the low socio-economic level of the family, the presence of more than four children in the family, the youth of the mother, less continuous supervision in the household, and the unsafe storage of medicines and household cleaning products [9–17]. Both prescription and over-the-counter (OTC) medicines may be responsible for poisonings [18, 19]. Burghardt et al. [20], in an ecological study of secondary data from the American Association of Poison Control Centers from 2000 through 2009, showed that an increase in the use of hypoglycaemic, antihyperlipidaemic, beta-blocking, and opioid drugs by adults was associated with an increase in the number of poisoned children visiting emergency rooms and admitted to hospital. Lovegrove et al. [21] conducted a study between 2004 and 2013 using data from the National Electronic Injury Surveillance System-Cooperative Adverse Drug Event Surveillance Project to assess trends in emergency department visits for unsupervised medication exposures in children aged < 6 years and found that easy access to medicines and lack of supervision at home are the main reasons for domestic poisoning of children. The objective of our study was to measure the prevalence of unsafe medicine storage in households with a 4-year-old child from the 2004 Pelotas Birth Cohort as a way of assessing the potential vulnerability of children to medicine poisoning.

## Methods

### Study design

Cross-sectional study nested in the 2004 Pelotas Birth Cohort.

### Setting and participants

The city of Pelotas is in southern Brazil and had a population of approximately 330,000 inhabitants in 2010 [22]. All live births in the city’s hospitals (*N* = 4231) in the year 2004 to mothers living in the urban area of Pelotas who agreed to participate were included in the 2004 Pelotas Birth Cohort Study. The participation refusal rate was less than 1%. These children were followed through household interviews at 3, 12, 24, and 48 months of age (4 years old). Information on the storage of medicines in the home at the 4-year follow-up was obtained from 3799 (92%) cohort participants. This cohort is being followed on a regular basis, and details about the study design and methodology can be found elsewhere [23].

### Variables and data sources/ measurement

The following questions were designed to ascertain whether medication was stored unsafely at home: *“Do you store medications: in the kitchen? (yes/no), in the bathroom? (yes/no), in the child’s bedroom? (yes/no), in another bedroom? (yes/no), in any other room?”*; *“Do you keep medications unlocked in a drawer or a cabinet?” (yes/no);* and for those mothers who answered yes, was asked:*“Are these drawers or cabinets installed at a height below the eye level of an average adult?” (yes/no).* Unsafe storage conditions were deemed to exist when medicines were kept in an unlocked drawer or cabinet and at a height below the eye level of an average adult [24].

The study explored a variety of independent variables. We assessed the following demographic information: the child’s sex; the age of the mother (categorized into 13–19, 20–29 and 30 or more years of age); and the self-reported skin colour of the mother (categorized into white, black, brown or mixed, Asian and Native). We assessed the following socioeconomic characteristics: the educational level of the mother and father (years of schooling categorized into 0–4, 5–8, 8–11, and 12 and more years); the employment status of the mother (actively working after the child was 2 years of age); the household’s socioeconomic status according to the Brazilian Association of Market Research Institutes [25], categorized into A/B, C and D/E, where A is the richest and E the poorest (we grouped together A/B and D/E in order to homogenize the size of the categories); and the marital status of the mother (living with a husband or partner). We reviewed the following variables regarding maternal and family characteristics: the identity of the child’s primary caregiver before age 4 years (relative or unrelated); the self-reported health of the mother (excellent/very good, good, and fair/poor); chronic conditions of the mother and father (mental illness, diabetes, or high blood pressure); and the number of people living in the household (2, 3, 4, 5 or more). Finally, we assessed the following variables regarding the child’s health care: breastfeeding duration (never breastfed, or breastfed 1–30, 31–180, 181–365, or over 365 days); private health insurance (yes/no); number of health care visits after the child was 2 years old (0, 1, 2, or 3 or more); utilization of emergency or hospital care services after 2 years old; history of poisoning involving medicines or household cleaning products after 2 years old (no/yes for medicines; no/yes for household cleaning products; no/yes for both); history of other injuries in the home from 2 to 4 years old (falls, cuts and burns); and child’s use of medications in the preceding 15 days.

### Statistical methods

**Fig. 1:**
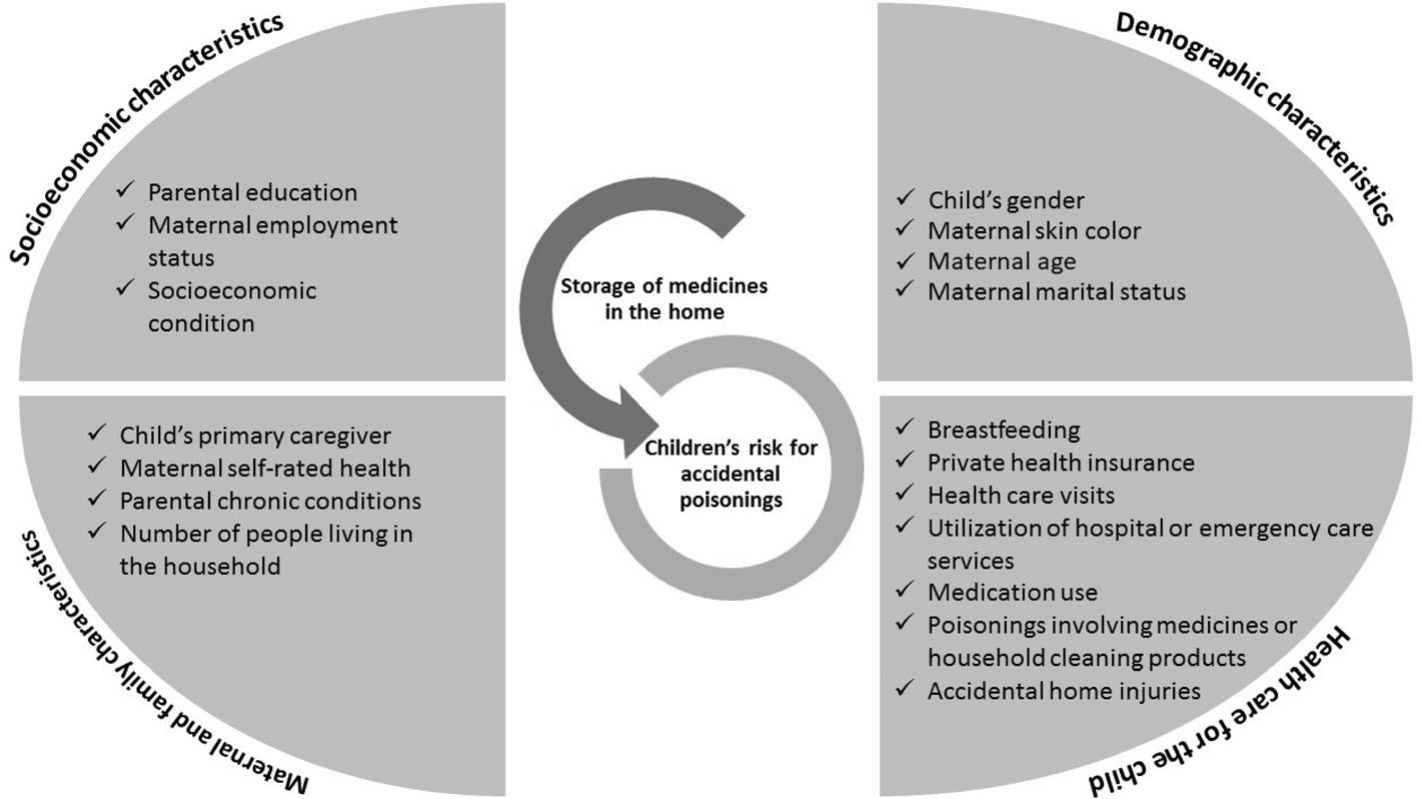
Theoretic model linking independent variables to the unsafe storage of household medicines

Statistical analyses were performed using Stata version 12.0 (Stata Corp., College Station, US. We carried out analyses, first to describe storage type and location of medicines in the household, presenting absolute and relative frequencies and prevalence of unsafe storage of medicines. Second, we calculate the characteristics of the sample (distribution) and third the frequency of the outcome (unsafe storage of medicines at home) according to the independent variables and the respective 95% confidence interval (95% CI). A theoretic model linking the independent variables to the unsafe storage of medicines in the household is shown in Fig. 1.

### Ethical considerations

The study was approved by the Research Ethics Committee of the Federal University of Pelotas Medical School (CEP/FAMED/UFPEL) under the number OF.012/07. Mothers who agreed to be interviewed were enrolled in the study after signing an informed consent document. Participants were advised that their participation was voluntary and that they could withdraw from the study at any time without the need to give a reason. Participants were also assured of the confidentiality of the information that they provided.

## Results

Of the 4231 children included in the original 2004 cohort, 4137 were able to participate at the age of 4 years (94 had died within 4 years of birth). After subtracting losses and refusals (*n* = 338), 3799 interviews were conducted that provided information about medicine storage in the household.

The most frequent storage locations were the kitchen (57.0%) and the child’s bedroom (27.4%), whereas the least frequent storage location was the bathroom (6.2%). A total of 3072 (80.9%) mothers or caretakers reported storing medicines in an unlocked drawer or cabinet, and 813 (26.5%) of them stored medicines in cabinets at a height below the eye level of an average adult. The prevalence of unsafe storage (medicines stored in an unlocked area and within the reach of children) was 21.4% (20.1–22.7%) (Table 1).

**Table 1:** Storage type and location of household medicines in families with children four-year-olds from the 2004 Pelotas Birth Cohort (Brazil)

Storage type and location	N	%
Storage locations		
Kitchen	2166	57.0
Bathroom	235	6.2
Child’s bedroom	1040	27.4
Other bedroom	985	25.9
Other room	255	6.7
Medicines stored unlocked in a drawer or a cabinet (*N* = 3797)*	3072	80.9
Medicine stored unlocked in a drawer or cabinets installed below eye level of an average adult (*N* = 3072)	813	26.5
Unsafe storage**(*N* = 3797*)	**813**	**21.4**

Detailed legend: * 2 missing (not answer)

**When medicines were kept in an unlocked drawer or cabinet and at a height below the eye level of an average adult

Total sample *N* = 3799

Table 2 shows the distribution of the sample and the proportion of unsafe storage of medicines in the home according to socioeconomic, demographic and health-related characteristics. Over 53% of the households were classified into socioeconomic group C. Most parents had five or more years of schooling, 35.2% of the families had five people or more living in the household, and 62% of the children were born to white mothers. The child’s primary caregiver was a relative in 75.5% of the families. Among the mothers, most (79.7%) were living with a partner, 65.3% of them were employed after the child was 2 years old, and approximately half of them (46.9%) self-rated their health as good. Over 50% of the parents did not report any chronic conditions. Unsafe medication storage was more frequent in families characterized by parents with limited education and a low socioeconomic status.

**Table 2:** Study sample and unsafe storage of household medicines in families with children four-year-olds from the 2004 Pelotas Birth Cohort (Brazil)

Variables	Study sample		Unsafe storage		
	N	%	N	%	95% CI
Child’s gender					
Male	1973	51.93	428	21.71	19.89–23.58
Female	1826	48.07	385	21.08	19.23–23.03
Maternal skin colour					
White	2329	61.97	508	21.81	20.15–23.55
Black	626	16.66	128	20.45	17.35–23.82
Brown or mixed	764	20.33	165	21.63	18.73–24.69
Asian or Native	39	1.04	5	12.82	4.29–27.43
Maternal age					
13–19	710	18.70	167	23.55	20.45–26.82
20–29	1878	49.46	380	20.23	18.44–22.12
30 or more	1209	31.84	266	22.02	19.70–24.44
Maternal education (years of schooling)					
0–4	581	15.36	115	19.83	16.63–23.27
5–8	1405	37.14	291	20.71	18.62–22.93
8–11	1341	35.45	285	21.27	19.09–23.54
12 or more	456	12.05	118	25.88	21.92–30.16
Paternal education (years of schooling)					
0–4	560	16.30	129	23.04	19.61–26.75
5–8	1470	42.78	299	20.35	18.31–22.59
8–11	1064	30.97	232	21.83	19.36–24.41
12 or more	342	9.95	79	23.10	18.74–27.94
Maternal employment status after the child was born					
No	1317	34.74	262	19.91	17.77–22.15
Yes	2474	65.26	548	22.15	20.53–23.84
Socioeconomic status (ABEP)					
A and B	974	25.92	227	23.31	20.68–26.09
C	1997	53.15	415	20.79	19.02–22.63
D and E	786	20.92	160	20.36	17.59–23.34
Primary caregiver					
Unrelated	925	24.50	217	23.46	20.76–26.32
Relative	2850	75.50	589	20.68	19.19–22.20
Maternal self-rated health					
Excellent / very good	1314	34.59	270	20.56	18.39–22.84
Good	1781	46.88	392	22.01	20.11–24.01
Fair / poor	704	18.53	151	21.48	18.47–24.67
Maternal chronic condition					
No	2080	54.75	449	21.59	19.84–23.42
Yes	1719	45.25	364	21.20	19.27–23.18
Paternal chronic condition					
No	2315	67.57	485	20.96	19.31–22.67
Yes	1111	32.43	249	22.43	19.99–24.98
Number of household members					
2	136	3.58	27	19.85	13.51–27.55
3	1068	28.11	253	23.69	21.17–26.36
4	1257	33.09	263	20.92	18.70–23.28
5 or more	1338	35.22	270	20.21	18.06–22.43

Detailed legend: Total sample N = 3799

Table 3 shows that 37% of the children were breastfed for 1–6 months and that only 6% were not breastfed at all. A history of injuries at home after 2 years old was reported for 2939 children (77.5%), 55.4% of the children did not attend any health care visits from 2 to 4 years old, and hospitalization or emergency care services utilization from 2 to 4 years old was reported for more than half of the children (58.2%). A total of 110 children (2.9%) experienced poisonings involving medicines (*n* = 71) or household cleaning products (*n* = 39) from 2 to 4 years old. Unsafe storage of medicines in the home was more frequent among children without private health insurance and who used medicines in the last 2 weeks.

**Table 3:** Unsafe storage of household medicines in families with children four-year-olds, child’s health and history of poisoning. 2004 Pelotas Birth Cohort (Brazil)

Variables	Study sample		Unsafe storage		
	N	%	N	%	95%CI
Breastfeeding duration (months)					
None	223	5.87	62	27.80	22.03–34.18
Less than one	210	5.53	54	25.71	19.95–32.18
1–6	1403	36.93	282	20.11	18.03–22.29
6–12	639	16.82	127	19.87	16.85–23.18
More than 12	1324	30.66	288	21.77	19.56–24.07
Private health insurance					
No	2242	59.08	462	20.62	18.95–22.34
Yes	1553	40.88	350	22.54	20.48–24.70
Number of health care visits					
0	2104	55.38	449	21.35	19.61–23.15
1	1140	30.01	237	20.79	18.47–23.26
2	431	11.35	98	22.74	18.86–26.99
3 or more	124	3.26	29	23.58	16.26–31.83
Hospitalization or use of emergency care services					
No	1587	41.80	328	20.68	18.70–22.75
Yes	2210	58.23	485	21.94	20.24–23.73
Medicines used in the 2 preceding weeks					
No	1943	51.16	371	19.10	17.37–20.91
Yes	1855	48.84	442	23.84	21.90–25.83
History of poisoning involving medicines or cleaning products					
No	3688	97.10	788	21.38	20.05–22.73
Yes, medicines	71	1.87	18	25.35	15.77–37.08
Yes, cleaning products	39	1.03	6	15.38	5.86–30.53
Prior history of home injuries					
No	855	22.54	179	20.94	18.25–23.82
Yes	2939	77.46	632	21.51	20.03–23.03

Detailed legend: Total sample *N* = 3799

## Discussion

This study found that the prevalence of unsafe storage of medicines was 21.4% (20.1–22.7%) in households with a 4-year-old child from the 2004 Pelotas Birth Cohort. This finding is lower that the results from another Brazilian study conducted of 280 households in São Paulo, Brazil, which found supplies of medicines in 255 (91%) households, of which 41.1% had unsafe/inadequate storage [26]. However consistent with finding from Tourinho et al. [27] that reported 22.4% of unsafe storage of household medicines in a study of 705 households from two cities in São Paulo, Brazil; that study involved a total of 2004 children and adolescents, of which 39.4% of the children were 6 years old or younger, and 91% of the households kept supplies of medicines at home.

Beirens et al [24] conducted a cross-sectional observational survey in the Netherlands using the same outcome definition as this study and obtained that 50.1% of the toddlers surveyed were exposed to unsafe storage of possible poisonous products in the home. Al Ruwaili et al, in a cross-sectional survey conducted in Saudi Arabia, found that 93% of children under 6 years old were at risk of accidental poisoning by medicines [28].

Studies performed in Parana State by Margonato et al. [14] collected data during home visits to patients with a record of acute unintentional drug poisoning according to the Poison Control Centre in Maringá and found that the majority of the victims were under 5 years old (59.7%), males (54.2%), and from lower-income groups (63.9%) and that there was a significant association (*p* < 0.05) between medicine poisoning in children and inadequate medicine storage. In Rio Grande do Sul State, Ramos et al. [17] surveyed similarly situated families and found that they are approximately 17 times more likely to have children poisoned by medicines. The study population in that case consisted of children aged from 0 to 14 years old who were victims of acute poisoning and who had been attended at an emergency unit in the period from 2008 to 2012. It is important to emphasize that the health of the mother is important for the health of the child. In this regard, a study conducted by Siqueira et al. [29] on maternal depression showed that it is a common condition associated with several child health outcomes and that the median number of injuries among boys was higher for those whose mothers consistently presented symptoms of depression. That study also showed that maternal depression is associated with a higher incidence of injury among children of both sexes aged 2–4 years, even after adjustment for confounding factors [29].

The prevalence of unsafe medicine storage observed in the current study is consistent with findings from other studies [30–32]. Groom et al. [33] conducted a cross-sectional study of children aged 0–4 years who were admitted to hospital due to unintentional poisoning in the former National Health Service Trent Region in the East Midlands, UK between 1 April 1995 and 31 March 1997 and showed that the number of hospital admissions due to medicine poisonings was 2–3 times higher for low-income children (IRR 2.49, 95% CI 1.87–3.30) and that those admissions were most commonly associated with accidental ingestion of benzodiazepines (IRR 5.63; 1.72–18.40), antidepressants (IRR 4.58; 1.80–11.66), and anti-cough medicines (IRR 3.93; 1.67–9.24).

Wealthier families whose children have a private health plan presented a lower rate of unsafe medicine storage. The impact of medicine on the healthcare of children under 4 years of age is well known. In this age group, with the cost of medicines in over 40% of families is the primary health expense, and the wealthier the family is, the higher the expenditure on medicines and private health plans [34]. In addition, Barcelos et al., 2017 [5], in a study using the same sample as our study, showed that adolescent mothers and poorer families with less education can benefit from preventive health measures to minimize risk injury to children.

The highest prevalence of unsafe storage of medicines was observed in households whose children have used medicines in the last 2 weeks, as well as in households where the parents had a lower level of education, which is consistent with the results obtained in a previous study [24] that found an association between lower level of education and unsafe storage of medicines.

Another important identification was the distribution of medicines in many rooms around the house. This lack of organization with medicines stored in multiple places, may lead to more opportunities for children to gain access to medicines. The families reported storing the medicines in different places, a fact that was also observed by other authors [24, 28]. However, Morrongiello et al. [35] and Gibbs et al. [36] showed adults may have inaccurate perceptions about whether a child has access to unsafely stored substances.

It should be taken into account that the results presented in this study are only descriptive. Studies aimed at understanding the risk factors related to unsafe medicine storage should include adjusted analysis for possible confounding factors.

Morrongiello et al. [37] further showed that parents intend by their reactions to reduce children’s injury-risk behaviours, but it study revealed some unexpected patterns that differ between boys and girls and relate to the frequency with which parents react by teaching or disciplining their children when they engage in risky behaviours.

The safe storage of medicines can mean that a child is safe at home. Although the results cannot be used in isolation to dictate clinical or regulatory actions, they have important practical implications for reducing poisoning in children. The results of this study represent the perspective of a medium-sized city in the southern region of the country. However, it does not limit the general applicability of the study; the results from studies of the Pelotas cohort are extremely similar to the national averages [38].

The results of this study are consistent with the initiatives adopted by the Office of Disease Prevention and Health Promotion Healthy People 2020 to reduce drug poisoning in children under the age of five by at least 10% by the end of the decade [39]. According to Budnitz et al. [8], meeting this objective will require a cooperative undertaking involving researchers, physicians, pharmacists, pharmaceutical companies, parents and caretakers. The importance of cooperation in preventing the poisoning of children can be seen in the PROTECT Initiative: Advancing Children’s Medication Safety, which has inspired the development of safety measures for medicines [40]. Studies have demonstrated that children gain access to medicines because the bottles are left open or are improperly closed or the medicines are put into other receptacles [41, 42]. According to Budnitz et al. [43] and Lovegrove et al. [21], the reasons for emergency room visits by poisoned children under 5 years old (95%) are the easy access to medicines and the absence of adult supervision at home. The significance of this problem lies mainly in the factors predisposing unintentional poisoning.

In a systematic review carried out by Barcelos et al. [44], educational interventions to reduce the risk factors and behaviours that cause childhood injuries were effective, suggesting the need for ongoing education of parents and caregivers regarding the risks for accidental medicine poisoning in young children. Substantial reductions in the risks and rates of injuries, as well as improvement in the knowledge of parents/caregivers and children regarding the prevention of accidents, were observed in the home [44].

In Brazil, unlike in other countries, most medicines are commercialized in blisters. In addition to the mandatory language in the medicine’s accompanying leaflet to “keep out of the reach of children”, the implementation of guidance that the medicine should be stored in a locked place or at a height above an adult’s head may contribute to keeping a child safe at home. Although the incidence of poisoning can be reduced by using child-resistant packaging, such packaging is not child-proof [45], and the research has demonstrated that up to 20% of child poisonings involved grandparents’ medication [46]. Soori et al. [47] concluded that adequate supervision was the most important factor that prevented childhood accidents, highlighting the need for adequate supervision of children under 5 years old. Parents and caregivers remain the first line of defence in preventing these incidents, and their behaviour is influenced by their knowledge and attitudes towards safe storage [48]. The research indicates that 1 in 3 parents believe that as long as a child is being watched, it is not important where medicine is stored [49]. Many parents overestimate their child’s ability to understand the potential dangers and follow safety rules, so supervision alone will not protect against medicine poisoning [49, 50].

Another important point that warrants discussion is the impact that the frequency of unsafe storage of household medicines has on the 4-year-old children from the 2004 cohort. An initial concern is the children’s vulnerability to medicines at home, which may lead to medicine poisoning. The National Network of Poisoning Control Centres (Rede Nacional de Centros de Controle de Intoxicações) in Brazil has reported medicines as the main agent responsible for poisoning events, even with all the advances in the toxicology field, the changes in packaging, and the educational messages about medicine safety [51]. In addition, according to the Centre of Toxicological Information of Rio Grande do Sul State (Centro de Informações Toxicológicas do Rio Grande do Sul) [52], in the period between 1980 and 2005, out of 62,071 incidents, 28.1% involved children younger than six years old, representing the second largest age group to suffer from poisoning. In 2013, medicine poisonings were the most common cases of poisoning, with 6036 visits to the Emergency Room of the Centre of Toxicological Information, representing 32.3% of all occurrences. Children under 5 years old represented 28% (*N* = 1692) of the incidents related to medicine poisoning. Seventy-six incidents were reported in the city of Pelotas for children under 5 years old, representing 4.5% of the medicine-related occurrences in the year 2013 in Rio Grande do Sul State [53]. The results highlight the challenge of identifying strategies that promote the safe storage of medicines, especially the adoption of protective measures in the everyday life of families. The children of working mothers had a lower risk of poisoning than the children of stay-at home mothers [54]. In addition, stay-at-home mothers provided inadequate supervision to their children and may have inadvertently created an unsafe home environment due to poor medicine storage habits [16]. Furthermore, the absence from the home of at least one parent was associated with an increased risk of accidental poisoning [16].

A systematic review that included 98 studies, involving more than 2 million people, evaluated the effectiveness of education about home safety and safety equipment in improving safety practices at home. Individual and face-to-face educational interventions effectively improved safe practices and were also found to effectively increase the safe storage of medicines (OR = 1.53; 95% CI 1.27–1.84) and promote the maintenance of ready access to the telephone number of the centre for poison control (OR = 3.30; 95% CI 1.70–6.39) [4].

To our knowledge, since no questionnaire has been validated for Brazil regarding the correct storage of medicines at home, we suggest that future research be conducted to validate a questionnaire to investigate safety practices of children at home through personal reports of parents or other adults responsible for the education of children under 5 years of age [54, 55]. There are criteria for the safe storage of medicines at home, and these criteria were used in this study.

## Conclusions

Research that identifies the behaviour of families concerning the storage of medicines supports the implementation of measures aimed at preventing harm. This is the first cohort study in Brazil that has investigated the unsafe storage of medicines at home. This study aims to contribute to the adoption of safety measures that incorporate the learnings from the study to develop educational interventions on the safe storage of medicines at home, thereby reducing the poisoning of children by household medicines.

The storage of medicines at home is an important public health issue. The results from our study indicate that medicines were unsafely stored in a 21.4% of homes, which can increase children’s vulnerability to poisoning from household medicines. To minimize the unsafe storage of medicines, education about the safe storage of medicines should be reinforced by health professionals.

## Data Availability

The dataset generated and/or analysed during the current study are not publicly available because it is extremely long and costly. Both in terms of money and time involved in project writing and data collection, which involves complex data and local specificities. Thus, responsible use of the data includes knowledge of the study design, objectives and of the local health system. However, the dataset can be available from the corresponding author on reasonable request.

## Abbreviations

ABEP: Brazilian Association of Research Companies
CEP/FAMED/UFPEL: Research Ethics Committee at Universidade Federal de Pelotas Medical School
CI: Confidence Interval
CNPq: The Brazilian National Council for Scientific and Technological Development
FAPERGS: Research Support Foundation of the State of Rio Grande do Sul, Brazil
IRR: Incidence Rate Ratio
N: Total sample
OTC: Over-the-counter
